# *Campylobacter concisus* upregulates PD-L1 mRNA expression in IFN-γ sensitized intestinal epithelial cells and induces cell death in esophageal epithelial cells

**DOI:** 10.1080/20002297.2021.1978732

**Published:** 2021-09-14

**Authors:** Seul A Lee, Fang Liu, Doo Young Yun, Stephen M Riordan, Alfred Chin Yen Tay, Lu Liu, Cheok Soon Lee, Li Zhang

**Affiliations:** a School of Biotechnology and Biomolecular Sciences, University of New South Wales, Sydney, Australia; b Gastrointestinal and Liver Unit,Prince of Wales Hospital, University of New South Wales, Sydney, Australia; c Helicobacter Research Laboratory, Marshall Centre for Infectious Diseases Research and Training, School of Pathology and Laboratory Medicine, University of Western Australia, Perth, Australia; d School of Medical Sciences, University of New South Wales, Sydney, Australia; e School of Medicine, Western Sydney University, Sydney, Australia; f South Western Sydney Clinical School, University of New South Wales, Sydney, Australia; g Central Clinical School, University of Sydney, Sydney, Australia; h Department of Anatomical Pathology, Liverpool Hospital, Sydney, Australia

**Keywords:** PD-L1, *Campylobacter concisus*, IFN-γ, intestinal epithelial cells, esophageal epithelial cells

## Abstract

**Introduction:** *Campylobacter concisus* is an oral bacterium that is associated with inflammatory bowel disease (IBD) and Barrett’s esophagus (BE). Programmed cell death ligand-1 (PD-L1) is an immune checkpoint protein that is used by tumor cells for immune evasion and has increased expression in patients with IBD and BE. We examined whether *C. concisus* upregulates PD-L1 expression in intestinal and esophageal epithelial cells.

**Methods:** Human intestinal epithelial HT-29 cells and esophageal epithelial FLO-1 cells with and without interferon (IFN)-γ sensitization were incubated with *C. concisus* strains. The level of PD-L1 mRNA was quantified using quantitative real-time PCR. Cytokines were measured using Enzyme-Linked Immunosorbent Assay (ELISA). Apoptosis of HT-29 and FLO-1 cells were measured using caspase 3/7 assay.

**Results:** We found that intestinal epithelial cells with IFN-γ sensitization incubated with *C. concisus* significantly upregulated PD-L1 expression and significantly increased the production of interleukin (IL)-8. Whereas, PD-L1 expression was significantly inhibited in IFN-γ sensitized FLO-1 cells incubated with *C. concisus* strains. Furthermore, FLO-1 cells with and without IFN-γ sensitization incubated with *C. concisus* strains both had significantly higher levels of cell death.

**Conclusion:** *C. concisus*has the potential to cause damage to both intestinal and esophageal epithelial cells, however, with different pathogenic effects.

## Introduction

*Campylobacter concisus* is a Gram-negative, spiral-shaped bacterium [1]. *C. concisus* normally colonizes the human oral cavity and behaves as a commensal oral bacterium [2]. However, the presence of *C. concisus* in other parts of the gastrointestinal tract is associated with gastrointestinal diseases [3]. A higher prevalence of *C. concisus* in the intestinal tract is associated with inflammatory bowel disease (IBD). IBD is a group of chronic inflammatory diseases of the gastrointestinal tract and a risk factor for colon cancer [4–6]. Crohn’s disease (CD) and ulcerative colitis (UC) are the two major clinical forms of IBD [7]. *C. concisus* has two genomospecies (GS) [8,9]. Studies found that *C. concisus* strains associated with IBD are GS2 strains [9–11].

*C. concisus* is also associated with esophageal diseases [12–14]. Barrett’s esophagus (BE) is a condition occurring at the lower esophagus, in which the normal squamous epithelium is replaced by intestinal-type columnar epithelium (intestinal metaplasia) [15,16]. BE is a complication of gastroesophageal reflux disease (GERD) and a risk factor for esophageal adenocarcinoma [17–19].

Programmed cell death ligand-1 (PD-L1) is a molecule that is expressed by hematopoietic cells and nonhematopoietic cells [20–23]. PD-L1 interacts with its receptor programmed cell death protein-1 (PD-1) expressed on activated T cells, leading to inhibition of T cells [24,25]. The physiological role of the PD-L1 and PD-1 pathway is to prevent excessive immune responses. Some tumor cells express PD-L1 on their surface to evade the attacks from cytotoxic T cells [25–27]. Increased expression of PD-L1 was also detected in intestinal tissues of patients with IBD and esophageal tissues of patients with BE [28–30]

Several cytokines upregulate the expression of PD-L1 such as interferon (IFN)-γ, tumor necrosis factor (TNF)-α, interleukin (IL)-1α and IL-17 [25,31–38]. In addition to cytokines, microbial factors were recently found to upregulate the epithelial expression of PD-L1 [39]. For example, *Helicobacter pylori*, a bacterium that causes chronic gastric inflammation, increases the gastric epithelial expression of PD-L1, and a commensal *Escherichia coli* strain K12 increases intestinal epithelial PD-L1 expression in IFN-γ sensitized intestinal epithelial cells [40–42].

We hypothesize that *C. concisus*, an oral bacterium that is associated with both IBD and BE, has the potential to upregulate the expression of PD-L1 in both intestinal and esophageal epithelial cells and examined this hypothesis using HT-29 and FLO-1 cells as intestinal and esophageal cell culture models, respectively. Interestingly, we found that *C. concisus* showed different pathogenic effects in intestinal and esophageal epithelial cells.

## Materials and methods

### Isolation of C. concisus strains from a patient with BE

Currently, *C. concisus* strains have not been isolated from patients with BE. We, therefore, isolated *C. concisus* strains from the saliva samples of a patient with BE in this study. Briefly, *C. concisus* isolation was conducted as previously described [43]. Given that previous studies have found that some individuals are colonized with multiple different *C. concisus* strains, multiple isolates were collected in this study [9,44]. The bacterial proteins were subjected to sodium dodecyl sulphate-polyacrylamide gel electrophoresis (SDS-PAGE) for the initial determination of the strains as previously described [44]. Isolates with identical SDS-PAGE pattern were defined as the same strain. The putative strains were subjected to genome sequencing for confirmation and identification of genomospecies as described in our previous study [45]. The raw reads were assembled using the St. Petersburg genome assembler to obtain the draft genomes (SPAdes, Ver. 3.15.2) [46]. Genomes of these strains were annotated using the combination of National Center for Biotechnology Information (NCBI) Prokaryotic Genome Annotation Pipeline, Prokka (Ver. 1.11) and Rapid Annotations using Subsystems Technology server (RAST, Ver. 2.0) [47,48]. Genomes assemblies were submitted to NCBI genome database under the Bioproject ID PRJNA735562, the genome accession numbers for *C. concisus* strains BEO1 and BEO2 were JAHKNB000000000 and JAHKNC000000000 respectively.

### C. concisus strains used in this study

A total of four *C. concisus* strains were used in this study. P2CDO4 and P15UCO-S2 were isolated from patients with IBD in our previous studies [49–51]. P2CDO4 has pICON plasmid and *csep1* gene, which were found to be associated CD, and P15UCO-S2 has pSma1 plasmid which was shown to be associated with severe UC [9,11,45]. Strains BEO1 and BEO2 were isolated in this study as described in the previous section.

### Maintenance of HT-29 and FLO-1 cells

Human intestinal epithelial cell line HT-29 (ATCC no. HTB-38) and esophageal adenocarcinoma epithelial cell line FLO-1 (ECACC 11012001) were used in this study. HT-29 cells were cultured in McCoy’s 5A medium (Invitrogen, CA, USA) supplemented with 10% fetal bovine serum (Global Life Sciences Solutions, Parramatta, Australia), 100 U/ml penicillin, and 100 U/ml streptomycin (Thermo Fisher Scientific, CA, USA). FLO-1 cells were cultured in Dulbecco’s Modified Eagle Medium (Invitrogen) supplemented with 10% fetal bovine serum (Global Life Sciences Solutions), 2 mM Glutamine (Thermo Fisher Scientific) and 100 U/ml penicillin, and 100 U/ml streptomycin (Thermo Fisher Scientific). The cells were maintained at 37°C in a humidified incubator containing 5% CO_2_.

### Measurement of PD-L1 expression

#### Quantitative real-time PCR

IFN-γ is known to upregulate PD-L1 expression in tumor tissues, IBD and BE [25,28–34,37,52]. Therefore, IFN-γ was used as the positive control and to sensitize both HT-29 and FLO-1 cells in this study. The concentration of IFN-γ used in this study and incubation time were established in our previous study [42].

HT-29 and FLO-1 cells were cultured in 6-well cell culture plates at a concentration of 2 × 10^6^/well in a complete medium alone or containing 50 ng/ml recombinant human IFN-γ (Roche, Mannheim, Germany) for 12 hours. HT-29 and FLO-1 cells with and without IFN-γ sensitization were incubated with *C. concisus* strains at a multiplicity of infection (MOI) of 10 and 100 for 4 hours. In order to investigate whether *C. concisus* strains isolated from a patient with BE (strains BEO1 and BEO2) upregulate PD-L1 mRNA expression in FLO-1 cells following a longer incubation period, FLO-1 cells with and without IFN-γ sensitization were also incubated with strains BEO1 and BEO2 for 24 hours at MOI 100. Cells were washed three times with Dulbecco’s phosphate-buffered saline (DPBS) before collecting for the assessment of PD-L1 expression at the mRNA level. Cells without IFN-γ sensitization and bacterial treatment were used as the negative control.

The levels of PD-L1 mRNA expression in HT-29 cells and FLO-1 cells were quantified using qRT-PCR. RNA was isolated from cells using TRIzol reagent (Invitrogen) following the manufacturer’s instructions. Total RNA (2 μg) was reverse transcribed to cDNA using the Tetro cDNA Synthesis kit (Bioline, NSW, Australia) according to the manufacturer’s instructions. The PD-L1 cDNA was then quantified using SYBR green (Bioline). Primers and the conditions were adapted from our previous study [42]. The levels of PD-L1 mRNA expression were normalized to the levels of glyceraldehyde 3-phosphate dehydrogenase (GAPDH) and expressed as the fold change relative to untreated cells using the comparative threshold cycle CT (2-^ΔΔCT^) method [53]. Reactions were performed in triplicate and the experiment was repeated three times.

### Enzyme-linked immunosorbent assay

The cell culture supernatants of HT-29 and FLO-1 cells treated in the sections above were collected and subjected for measurement of five pro-inflammatory cytokines, including IL-1β, IL-18, IL-6, IL-8, and TNF-α using commercially available ELISA kits (Invitrogen), following the manufacturer’s instructions.

### Measurement of cell death

Caspase 3/7 activities in HT-29 and FLO-1 cells incubated with *C. concisus* strains at MOI 10 and 100 were measured to investigate whether *C. concisus* strains induces cell death after 4 hours. In addition, caspase 3/7 activities in FLO-1 cells with and without IFN-γ sensitization incubated with *C. concisus* strains BEO1 and BEO2 at MOI 100 after 24 hours were also measured. Cells (1 × 10^5^/well) were first seeded on black-walled 96-well plates with transparent bottoms for 1 day. Cells were incubated with *C. concisus* strains at MOI 10 and 100 for 4 hours. For 24 hours, FLO-1 cells with and without IFN-γ sensitization were incubated with BEO1 and BEO2 at MOI 100. Cells without bacterial treatment were used as the negative control and staurosporine-treated (1 μM; Sigma-Aldrich) cells were used as the positive control [54,55]. Caspase 3/7 activities were then measured using the CellEvent Caspase-3/7 Green ReadyProbes reagent (Invitrogen) according to the manufacturer’s instructions. The levels of active caspase 3/7 activity were expressed as fold change relative to the negative control.

### Measurement of C. concisus adherence to FLO-1 cells

*C. concisus* was previously found to adhere to HT-29 cells [56]. The adherence of *C. concisus* to FLO-1 cells was examined in this study using a previously described gentamicin assay [56]. FLO-1 cells were cultured with and without IFN-γ sensitization as described above, then were incubated with *C. concisus* strains BEO2 or P2CDO4 at MOI 100. Following 24 hours incubation, FLO-1 cells were washed three times with DPBS and incubated with 200 µg/ml gentamicin (Thermo Fisher Scientific) for 1 hour. Cells were then lysed with 1% Triton X-100 and placed onto HBA plates to grow for 24 hours. The CFU accounts of the same *C. concisus* strains in FLO-1 cells with and without IFN-γ sensitization were compared.

## Statistical analysis

One-way analysis of variance (ANOVA) with Dunnett’s test was performed to compare the levels of PD-L1 mRNA, IL-8 concentrations, and caspase 3/7 activities between different samples. *P* values less than 0.05 were considered as statistically significant.

## Results

### Isolation and genome information of C. concisus strains BEO1 and BEO2 from saliva samples of a patient with BE

Protein profile analysis of 20 *C. concisus* isolates revealed two different patterns, showing that this patient was colonized by two different *C. concisus* strains. Two strains (BEO1 and BEO2), one from each of the protein profiles, were used for genome sequencing and experiments in this study (Supplementary Figure 1). The genome sizes of strains BEO1 and BEO2 were 1.86 and 1.77 Mb, respectively. BEO1 had 93 contigs and BEO2 had 95 contigs with a fold coverage of 107.26× and 195.66×, respectively. N50 for BEO1 was 37,628 bp and for BEO2 was 29,126 bp. Both strains belonged to GS2.

### C. concisus upregulated PD-L1 expression in IFN-γ sensitized HT-29 cells but not in FLO-1 cells

**Figure 1.**
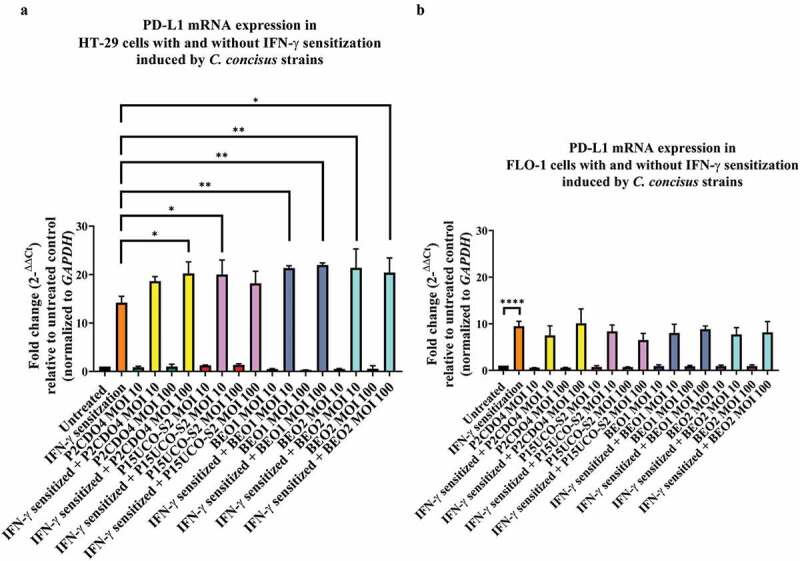
**The effects of *C. concisus* strains on PD-L1 mRNA expression in HT-29 cells and FLO-1 cells after 4 hours**. HT-29 cells **(A)** or FLO-1 cells **(B**) with and without IFN-γ sensitization were incubated with *C. concisus* strains (P2CDO4, P15UCO-S2, BEO1 or BEO2) at MOI 10 or 100 for 4 hours. PD-L1 mRNA expressions were measured by qRT-PCR. IFN-γ sensitized HT29 cells were used as the positive control and untreated cells were used as the negative control. The straight-line bar indicates significance test compared with IFN-γ sensitized cells. One-way analysis of variance (ANOVA) with Dunnett’s test was performed. Graphs are representative of averages of triplicate experiments ± standard error (* = *P* < 0.05; ** = *P* < 0.01; **** = *P* < 0.0001 indicates statistical significance). MOI: multiplicity of infection

*C. concisus* alone did not affect PD-L1 expression in both HT-29 cells and FLO-1 cells following 4 hours of incubation. The mRNA levels of PD-L1 in both HT-29 cells and FLO-1 cells incubated with four *C. concisus* strains at MOI 10 and MOI 100 did not show significant differences in comparison to cells without any treatment (*P* > 0.05) (Figure 1A and 1B).

In HT-29 cells sensitized with IFN-γ, *C. concisus* strains P15UCO-S2, BEO1 and BEO2 at MOI 10 induced a significantly higher expression of PD-L1 mRNA compared to IFN-γ sensitized HT-29 cells without any bacterial treatment (*P* < 0.05, *P* < 0.01, and *P* < 0.01 respectively). The fold changes of PD-L1 mRNA levels in cells incubated with these strains were 20.01 ± 2.12, 21.33 ± 0.34 and 21.39 ± 2.78, respectively (Figure 1A). At MOI 100, strains P2CDO4, BEO1 and BEO2 induced a significantly higher expression of PD-L1 in IFN-γ sensitized HT-29 cells compared to IFN-γ sensitized HT-29 cells without any bacterial treatment (*P* < 0.05, *P* < 0.01, and *P* < 0.05 respectively). The fold changes of PD-L1 mRNA levels in HT-29 cells incubated with these strains were 20.25 ± 1.68, 21.98 ± 0.30 and 20.38 ± 2.16, respectively (Figure 1A).

In FLO-1 cells, IFN-γ sensitization significantly increased PD-L1 mRNA expression (*P* < 0.0001) (Figure 1B). However, *C. concisus* strains did not significantly affect the PD-L1 mRNA expression in IFN-γ sensitized FLO-1 cells. The levels of PD-L1 mRNA in IFN-γ sensitized FLO-1 cells incubated with four *C. concisus* strains at MOI 10 and MOI 100 were not significantly different from IFN-γ sensitized FLO-1 cells without bacterial treatment (*P* > 0.05).

*C. concisus* strains did not induce apoptosis in both HT-29 cells and FLO-1 cells following 4 hours of incubation. The caspase 3/7 activities in HT-29 cells and FLO-1 cells did not show a significant change after 4 hours of incubation with the four *C. concisus* strains (Supplementary Figure 2).

### C. concisus *induced IL-8 production in HT-29 cells but not in FLO-1 cells*

**Figure 2.**
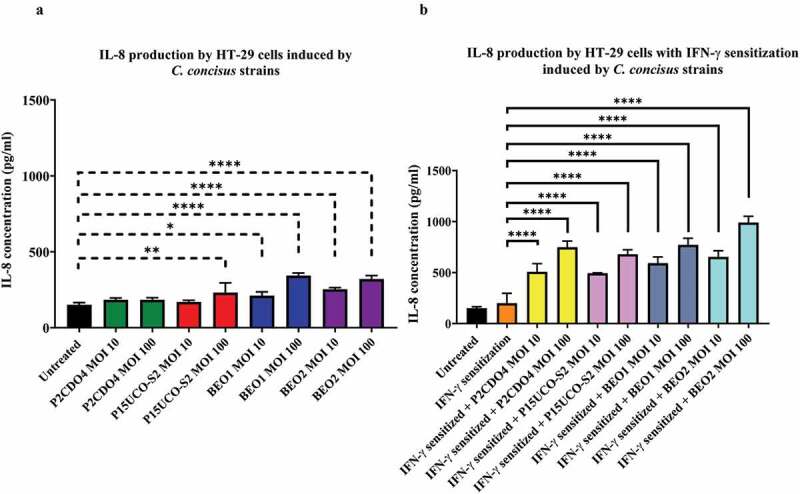
**IL-8 production by HT-29 cells with and without IFN-γ sensitization induced by *C. concisus* strains after 4 hours.**Concentrations of IL-8 in the cell culture supernatants of HT-29 cells without **(A)** and with IFN-γ **(B)** sensitization were measured by ELISA after 4 hours of incubation with *C. concisus* strains (P2CDO4, P15UCO-S2, BEO1 or BEO2). The dash-line bar indicates significance test compared with untreated HT-29 cells and the straight-line bar indicates significance test compared with IFN-γ sensitized HT-29 cells. One-way analysis of variance (ANOVA) with Dunnett’s test was performed. Graphs are representative of averages of triplicate experiments ± standard error (* = *P* < 0.05; ** = *P* < 0.01; **** = *P* < 0.0001 indicates statistical significance). MOI: multiplicity of infection

The IL-8 concentrations in the supernatants of HT-29 cells incubated with *C. concisus* strains P2CDO4 and P15UCO-S2 at MOI 10 and P2CDO4 at MOI 100 for 4 hours were 183.78 ± 6.45, 169.71 ± 6.19 and 183.84 ± 7.66 pg/ml, respectively. These concentrations were higher than the IL-8 concentration of the untreated HT-29 cells (151.12 ± 5.77 pg/ml), however, it did not reach a significant difference (*P* > 0.05) (Figure 2A). HT-29 cells incubated with BEO1 and BEO2 at MOI 10 induced a significantly higher production of IL-8 as compared to untreated HT-29 cells. The concentrations of HT-29 cells incubated with BEO1 and BEO2 at MOI 10 were 211.25 ± 14.30 and 253.30 ± 6.04 pg/ml respectively (*P* < 0.05 and *P* < 0.0001 respectively). At MOI 100, strains P15UCO-S2, BEO1 and BEO2 induced a significantly higher production of IL-8 as compared to untreated HT-29 cells, with the concentrations being 231.11 ± 37.20, 343.24 ± 9.85, and 320.58 ± 13.06 pg/ml, respectively (*P* < 0.01, *P* < 0.0001 and *P* < 0.0001 respectively). We also measured IL-1β, IL-18, IL-6, and TNF-α but the levels of these cytokines were below the detection level (data not shown).

IFN-γ sensitization has greatly enhanced the response of HT-29 cells to *C. concisus* in the production of IL-8. In IFN-γ sensitized HT-29 cells, the IL-8 concentrations in the supernatants of HT-29 cells incubated with *C. concisus* strains P2CDO4, P15UCO-S2, BEO1 and BEO2 at MOI 10 were 507.38 ± 46.47, 492.61 ± 3.88, 591.32 ± 36.05 and 653.39 ± 34.92 pg/ml respectively, which were significantly different from that in the supernatant of IFN-γ sensitized HT-29 cells without bacterial treatment (*P* < 0.0001) (Figure 2B). The levels of IL-8 induced by the four *C. concisus* strains at MOI 100 were even higher, being 748.26 ± 34.90, 679.25 ± 25.46, 770.98 ± 37.99 and 989.43 ± 36.35 pg/ml respectively (*P* < 0.0001) (Figure 2B). The levels of IL-1β, IL-18, IL-6, and TNF-α in IFN-γ sensitization with and without bacterial treatment were below deletion levels (data not shown). *C. concisus* strains did not induce the production of the cytokines measured in this study in FLO-1 cells, with or without IFN-γ sensitization.

### C. concisus *strains BEO1 and BEO2 induced cell death in FLO-1 cells after 24 hours of incubation*

Given that *C. concisus* strains did not show significant effects on PD-L1 expression, cytokine production and apoptosis in FLO-1 cells after 4 hours of incubation, we conducted further experiments to investigate these effects after 24 hours of incubation of *C. concisus* with FLO-1 cells. For these experiments, strains BEO1 and BEO2 at MOI 100 were used to incubate FLO-1 cells.

**Figure 3.**
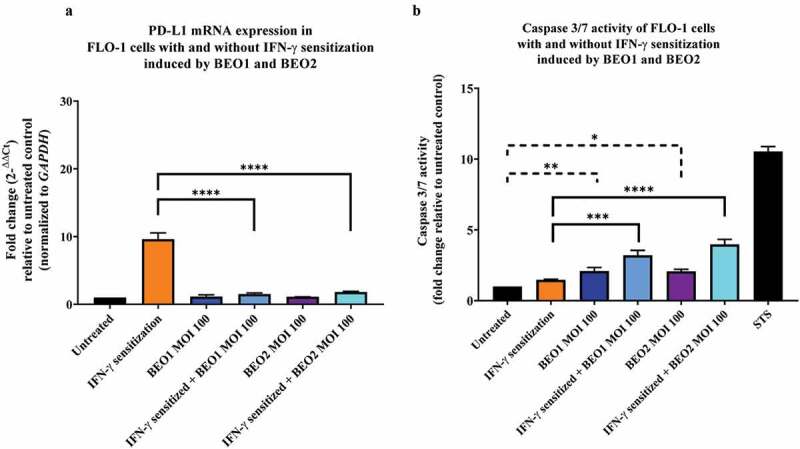
**PD-L1 mRNA expression and caspase 3/7 activities in FLO-1 cells with and without IFN-γ sensitization induced by *C. concisus* BEO1 and BEO2 at MOI 100 after 24 hours. (A)** The mRNA levels of PD-L1 in FLO-1 cells were measured using qRT-PCR. **(B)** The apoptotic effect of BEO1 and BEO2 on FLO-1 cells were determined by measuring caspase 3/7 levels using CellEvent™ caspase 3/7 green detection reagent. Staurosporine (STS) treated FLO-1 cells were used as the positive control. One-way analysis of variance (ANOVA) with Dunnett’s test was performed. Graphs are representative of averages of triplicate experiments ± standard error (* = *P* < 0.05; ** = *P* < 0.01; *** = *P* < 0.001 **** = *P* < 0.0001 indicates statistical significance). MOI: multiplicity of infection

*C. concisus* strains BEO1 and BEO2 alone did not significantly affect PD-L1 expression in FLO-1 cells after 24 hours of incubation. The levels of PD-L1 mRNA in FLO-1 cells without IFN-γ sensitization incubated with *C. concisus* strains BEO1 and BEO2 for 24 hours were not significantly different from that in FLO-1 cells without bacterial treatment (*P* > 0.05). (Figure 3A). In IFN-γ sensitized FLO-1 cells, both *C. concisus* strains significantly inhibited the level of PD-L1 induced by IFN-γ. The mRNA level of PD-L1 in IFN-γ sensitized FLO-1 cells incubated with BEO1 was 1.52 ± 0.08, which was significantly lower than the IFN-γ sensitized FLO-1 cells without bacterial treatment (9.61 ± 0.65) (*P* < 0.0001). The mRNA level of PD-L1 in IFN-γ sensitized FLO-1 cells incubated with BEO2 was 1.82 ± 0.05, which was also significantly lower than the IFN-γ sensitized FLO-1 cells without bacterial treatment (*P* < 0.0001) (Figure 3A).

Interestingly, while *C. concisus* strains BEO1 and BEO2 inhibited PD-L1 expression in IFN-γ sensitized FLO-1 cells, they induced apoptosis in these cells. The levels of caspase 3/7 activity in IFN-γ sensitized FLO-1 cells incubated with BEO1 and BEO2 were 3.20 ± 0.20 and 3.98 ± 0.25 respectively which were significantly higher than that of the IFN-γ sensitized FLO-1 cells (*P* < 0.001 and *P* < 0.0001 respectively) (Figure 3B). Furthermore, FLO-1 cells incubated with BEO1 (2.05 ± 0.14) and BEO2 (2.07 ± 0.10) had significantly higher levels of caspase 3/7 activity than that of the untreated cells (*P* < 0.01 and *P* < 0.05 respectively). There was no detectable cytokine production in the supernatants of FLO-1 cells incubated with strains BEO1 and BEO2 after 24 hours.

### IFN-γ sensitization increased C. concisus adhesion to FLO-1 cells

Both *C. concisus* strains BEO2 and P2CDO4 significantly increased adhesion to IFN-γ sensitized FLO-1 cells as compared to FLO-1 cells without IFN-γ sensitization (*P* < 0.05 and *P* < 0.01 respectively). In both strains, the adhesion increased more than two folds in IFN-γ-sensitized FLO-1 cells (Supplementary Figure 3).

## Discussion

In this study, we examined the effects of *C. concisus* strains on PD-L1 mRNA expression, pro-inflammatory cytokine production and caspase 3/7 activities in intestinal and esophageal epithelial cells using HT-29 and FLO-1 as the cell culture models.

In a local environment without the presence of IFN-γ, the predominant role of *C. concisus* on intestinal epithelial cells was to induce acute inflammation. Production of IL-8 by HT-29 cells was observed after a short period (4 hours) of incubation with *C. concisus* but PD-L1 mRNA expression was not significantly changed (Figure 1A and 2A). Sufficient numbers of bacteria are required to induce the production of IL-8 in HT-29 cells, as *C. concisus* strains induced a significantly higher IL-8 production at MOI 100 (Figure 2A). IL-8 is a key inflammatory cytokine involved in the recruitment of neutrophils to the infection site [57]. These results show that *C. concisus* has the potential to induce acute inflammation in the intestinal tract without the need of establishing long-term enteric colonization as long as sufficient *C. concisus* bacterial cells are translocated to the intestinal tract from the oral cavity. *C. concisus* is a spiral to curved shape microorganism with a flagellum and this structure may have provided the bacteria capability to cross the intestinal mucus layer and contact epithelial cells [1,56].

*C. concisus* strains induced a higher IL-8 production and significantly upregulated the expression of PD-L1 mRNA in IFN-γ sensitized intestinal epithelial cells. The threshold of bacterial numbers required for *C. concisus* to induce epithelial production of IL-8 in IFN-γ sensitized HT-29 cells was greatly reduced as compared to HT-29 cells without IFN-γ treatment. All *C. concisus* strains induced a significantly higher production of IL-8 at MOI 10 and the levels of IL-8 induced by *C. concisus* strains in IFN-γ sensitized HT-29 cells were greatly higher than that in HT-29 cells with IFN-γ sensitization (Figure 2). A previous study has shown that IFN-γ upregulates intracellular Toll-like receptor-4-MD-2 complex, which may have played a part in contributing to the upregulated response of IL-8 production induced by *C. concisus* in IFN-γ sensitized HT-29 cells [58]. Interestingly, *C. concisus* strains also significantly upregulated the expression of PD-L1 mRNA in IFN-γ sensitized HT-29 cells. PD-L1 is an immune checkpoint protein and the binding of PD-L1 to its receptor on T cells delivers an inhibitory signal, which is a mechanism by which the immune system limits immune-mediated tissue damages [24,25,35]. In chronic inflammatory conditions such as IBD, PD-L1 expression including epithelial PD-L1 expression is upregulated [28]. Prolonged upregulation of PD-L1 expression provides opportunities for pre-cancer and cancer cells to evade the attacks from the immune system, which may contribute to an increased risk of developing cancer associated with chronic inflammation. Our findings in this study suggest that in patients with chronic enteric inflammation such as IBD, IBD-associated oral bacterium *C. concisus* may further increase the enteric inflammation via enhancing the innate inflammatory immune response and also have the potential to inhibit the functions of effector T cells via upregulating epithelial PD-L1. In this study, we have examined PD-L1 mRNA expression. Future studies examining the effects of *C. concisus* on PD-L1 protein expression in IFN-γ sensitized HT-29 cells and the induced PD-L1 protein on the functions of effector T cells such as proliferation, cytokine productions, and cytotoxicity could further explore the possible role of *C. concisus* in immune evasion.

The effects of *C. concisus* on esophageal cells were different from that on intestinal epithelial cells. *C. concisus* did not induce production of IL-8 in FLO-1 cells following 4 hours of incubation at both MOI 10 and MOI 100. The bacterium also did not significantly affect PD-L1 mRNA expression in FLO-1 cells with and without IFN-γ sensitization after 4 hours of incubation (Figure 1B). *C. concisus* did not significantly change the caspase 3/7 activities in both FLO-1 cells and HT-29 cells after 4 hours of incubation. Therefore, apoptosis was not the reason why *C. concisus* did not significantly affect PD-L1 mRNA expression in FLO-1 cells after 4 hours (Supplementary Figure 2). Following incubation of *C. concisus* with FLO-1 cells with and without IFN-γ sensitization for 24 hours, there was still no IL-8 production by FLO-1 cells. We also measured IL-1β, IL-18, IL-6, and TNF-α and these were all below the detection level (data not shown). It appears that FLO-1 cells do not secret these common pro-inflammatory cytokines in response to *C. concisus* incubation. Interestingly, *C. concisus* significantly inhibited PD-L1 mRNA expression in IFN-γ sensitized FLO-1 cells (Figure 3A). Such a reduction of PD-L1 was due to cell death induced by *C. concisus* (Figure 3B). Previous studies detected increased *C. concisus* in esophageal tissues and increased cytokines [12–14]. Our finding that *C. concisus* induced apoptosis in FLO-1 with and without IFN-γ sensitization suggests that *C. concisus* may contribute to esophageal disease by damaging the epithelial barrier. Interestingly, such damage requires *C. concisus* to contact esophageal cells for sufficient time as apoptosis occurred in FLO-1 cells incubated with *C. concisus* for 24 hours but not in FLO-1 cells incubated with *C. concisus* for 4 hours. IFN-γ sensitization of FLO-1 cells resulted in a two-fold increase of *C. concisus* adhesion but no invasion was observed (Supplementary Figure 3), further supporting that adhesion of *C. concisus* to FLO-1 cells is required for causing epithelial damage.

Cell lines including HT-29 and FLO-1 have been used as *in vitro* models in this study. While cell line models provide the ease of cultivation and the consistency of results produced, they are tumor-derived cells and lack of the normal architecture of intestinal or esophageal epithelium. Future studies using human intestinal and esophageal organoids may provide further information more resembling the human physiological conditions.

In summary, in this study, we found that *C. concisus* caused different pathogenic effects in intestinal and esophageal epithelial cells. *C. concisus* induced IL-8 production in intestinal epithelial HT-29 cells without IFN-γ sensitization. In IFN-γ sensitized HT-29 cells, induction of IL-8 by *C. concisus* was enhanced and PD-L1 mRNA expression was upregulated. *C. concisus* induced apoptosis in esophageal epithelial FLO-1 cells and IFN-γ sensitization enhanced *C. concisus* induced cell death in FLO-1 cells. These data suggest a role that *C. concisus* has the potential to cause damage to both intestinal and esophageal epithelial cells, however, with different pathogenic effects.

## Supplementary Material

Supplemental Material
