# Supplementary material for: Campylobacter concisus upregulates PD-L1 mRNA expression in IFN-γ sensitized intestinal epithelial cells and induces cell death in esophageal epithelial cells
Source: J Oral Microbiol. 2021 Sep 14;13(1):1978732. doi: 10.1080/20002297.2021.1978732 (PMC8451702; doi:10.1080/20002297.2021.1978732)
Supplement: Supplemental Material [file ZJOM_A_1978732_SM2017.zip › Supplementary files/figure caption.docx]

**Supplementary Figure 1 Protein profiles for *C. concisus* BEO1 and BEO2.** Bacterial whole cell lysates were prepared by three freeze-thaw cycles, and 20 µg of proteins were subjected to sodium dodecyl-sulfate polyacrylamide gel electrophoresis. Protein bands unique to BEO1 or BEO2 are indicated with arrows.

**Supplementary Figure 2 Caspase 3/7 activities in HT-29 and FLO-1 cells following 4 hours of *C. concisus* treatment.** HT-29 cells **(A)** or FLO-1 **(B)** cells were treated with *C. concisus* strains (P2CDO4, P15UCO-S2, BEO1 or BEO2) for 4 hours at MOI 10 or 100. Staurosporine (STS) treated HT-29 cells or FLO-1 cells were used as the positive control. MOI: multiplicity of infection. One-way analysis of variance (ANOVA) with Dunnett's test was performed. Graphs are representative of averages of triplicate experiments ± standard error (**** = *P* < 0.0001 indicates statistical significance).

**Supplementary Figure 3 Adhesion of *C. concisus* strains on FLO-1 cells with and without IFN-γ sensitization.** FLO-1 cells with or without IFN-γ sensitization were treated with *C. concisus* strains P2CDO4 or BEO2 at MOI of 100 for 24 hours. The levels of bacterial adhesion were determined by gentamicin assay. One-way analysis of variance (ANOVA) with Dunnett's test was performed. Graphs are representative of averages of triplicate experiments ± standard error (* = *P* < 0.05; ** = *P* < 0.01 indicates statistical significance). MOI: multiplicity of infection.
