# Supplementary figures and images for: Campylobacter concisus upregulates PD-L1 mRNA expression in IFN-γ sensitized intestinal epithelial cells and induces cell death in esophageal epithelial cells
Source: J Oral Microbiol. 2021 Sep 14;13(1):1978732. doi: 10.1080/20002297.2021.1978732 (PMC8451702; doi:10.1080/20002297.2021.1978732)

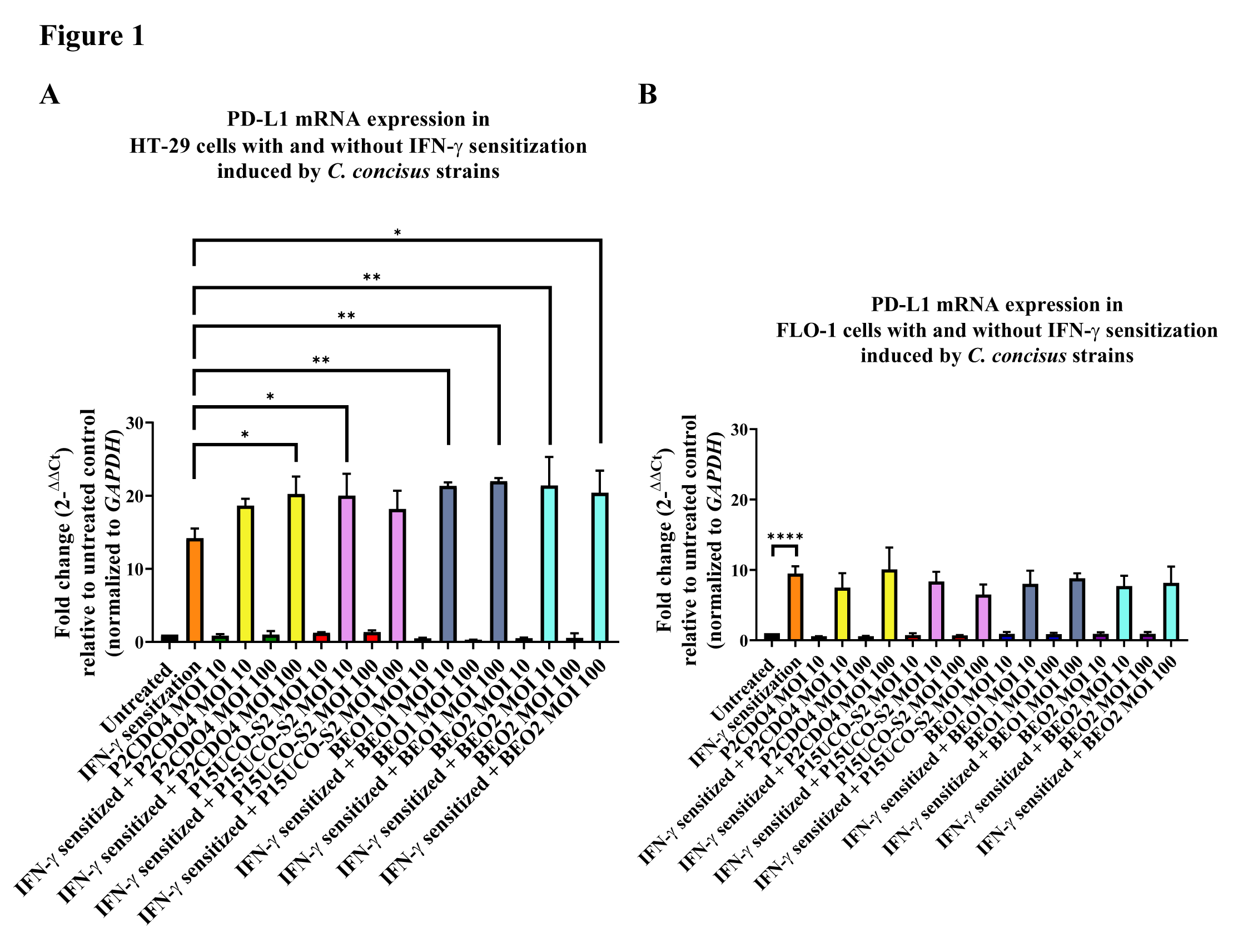

Supplement: Supplemental Material [file ZJOM_A_1978732_SM2017.zip › Supplementary files/Supplementary Figure 1.tif]

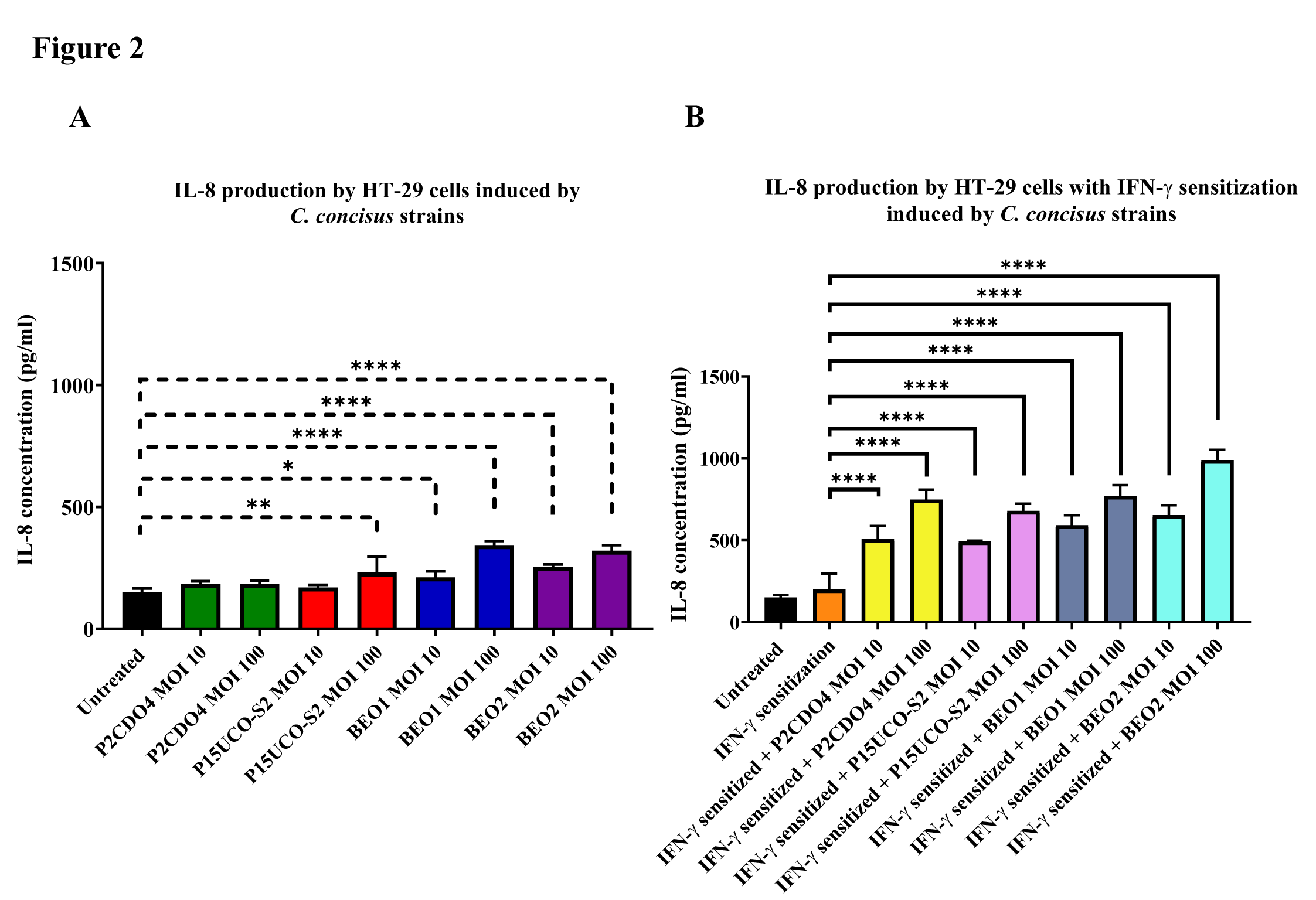

Supplement: Supplemental Material [file ZJOM_A_1978732_SM2017.zip › Supplementary files/Supplementary Figure 2.tif]

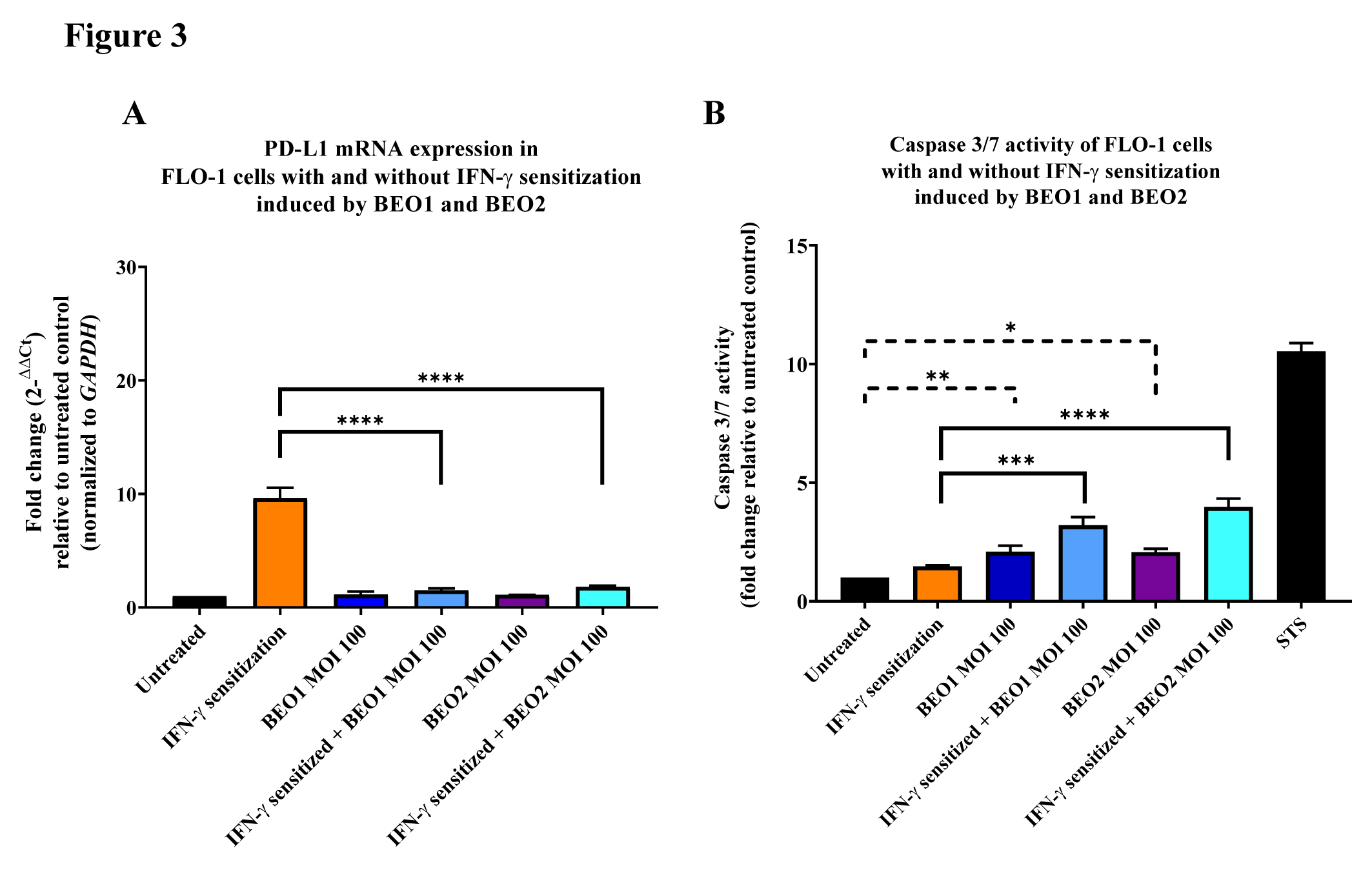

Supplement: Supplemental Material [file ZJOM_A_1978732_SM2017.zip › Supplementary files/Supplementary Figure 3.tif]
